# The *Fusarium graminearum* Histone Acetyltransferases Are Important for Morphogenesis, DON Biosynthesis, and Pathogenicity

**DOI:** 10.3389/fmicb.2018.00654

**Published:** 2018-04-26

**Authors:** Xiangjiu Kong, Anne D. van Diepeningen, Theo A. J. van der Lee, Cees Waalwijk, Jingsheng Xu, Jin Xu, Hao Zhang, Wanquan Chen, Jie Feng

**Affiliations:** ^1^State Key Laboratory for Biology of Plant Diseases and Insect Pests, Institute of Plant Protection, Chinese Academy of Agricultural Sciences, Beijing, China; ^2^Biointeractions & Plant Health, Wageningen Plant Research, Wageningen, Netherlands

**Keywords:** *Fusarium graminearum*, histone acetyltransferase, secondary metabolism, deoxynivalenol, pathogenicity

## Abstract

Post-translational modifications of chromatin structure by histone acetyltransferase (HATs) play a central role in the regulation of gene expression and various biological processes in eukaryotes. Although HAT genes have been studied in many fungi, few of them have been functionally characterized. In this study, we identified and characterized four putative HATs (*FgGCN5, FgRTT109, FgSAS2, FgSAS3*) in the plant pathogenic ascomycete *Fusarium graminearum*, the causal agent of *Fusarium* head blight of wheat and barley. We replaced the genes and all mutant strains showed reduced growth of *F. graminearum*. The Δ*FgSAS3* and Δ*FgGCN5* mutant increased sensitivity to oxidative and osmotic stresses. Additionally, Δ*FgSAS3* showed reduced conidia sporulation and perithecium formation. Mutant Δ*FgGCN5* was unable to generate any conidia and lost its ability to form perithecia. Our data showed also that *FgSAS3* and *FgGCN5* are pathogenicity factors required for infecting wheat heads as well as tomato fruits. Importantly, almost no Deoxynivalenol (DON) was produced either in Δ*FgSAS3* or Δ*FgGCN5* mutants, which was consistent with a significant downregulation of TRI genes expression. Furthermore, we discovered for the first time that *FgSAS3* is indispensable for the acetylation of histone site H3K4, while *FgGCN5* is essential for the acetylation of H3K9, H3K18, and H3K27. H3K14 can be completely acetylated when *FgSAS3* and *FgGCN5* were both present. The RNA-seq analyses of the two mutant strains provide insight into their functions in development and metabolism. Results from this study clarify the functional divergence of HATs in *F. graminearum*, and may provide novel targeted strategies to control secondary metabolite expression and infections of *F. graminearum*.

## Introduction

In different organisms, ranging from yeast to plants and animals, the interaction between four types of histones (H3, H4, H2A, H2B) tightly wrap the DNA in the nucleus in so-called nucleosomes (Kornberg and Lorch, 1999). These nucleosomes are the focal points of transcription control including gene activation and repression at the level of individual promoters, transcription units and whole chromosomal domains. Acetylation of histone, which was first reported in 1964 (Allfrey et al., 1964), is an important post-translational modification and regulation of major regulator of gene expression.

Histone acetylation is a reversible dynamic process. Hyperacetylation of histones results in a relaxed chromatin structure (euchromatin) and gene activation transcription through neutralizing positively charged lysine residues and weakening the interactions between histones and DNA, while hypoacetylation of histones can result in transcriptional repression by condensing the chromatin (heterochromatin) and limiting the accessibility of transcription factors to bind to DNA (Wang et al., 1998; Strahl and Allis, 2000). Lysine acetylation of histone H3 and H4 is the best studied in all the modifications described to date (Fischle et al., 2003). Histone acetylation is controlled by the opposite actions of the histone acetyltransferase (HAT) and histone deacetylase (HDAC) superfamilies. The balancing action of the two enzyme families is important for proper cell function and development (Lee and Workman, 2007). Characterizing the function of enzymes that regulate acetylation is an important way to elucidate the key role of post-translational modifications.

Histone acetylation occurs at multiple lysine residues and is usually carried out by the protein complex containing histone acetyltransferase (Wapenaar and Dekker, 2016). Histone acetyltransferases can be divided into nuclear A-type HATs and cytoplasmic B-type HATs, depending on the binding site in the cell. Based on sequence conservation within the HAT domain, A-type HATs can be classified into four different families, that is (i) GNAT (general control nonderepressible 5 (GCN5)-related acetyltransferase), (ii) MYST (named after the abbreviations of the founding members MOZ, Ybf2/Sas3, Sas2, and TIP60), (iii) p300/CBP (two human paralogs p300 and CBP, metazoan-specific), and (iv) Rtt109 (regulator of Ty1 transposition gene product 109, fungal-specific, a structural homolog of p300/CBP). In this study we focus on these A-type HATs.

GCN5 is a typical representative of the GANT family. GCN5 was initially named for the general regulation of amino acid synthesis signaling pathway in yeast (Hinnebusch and Fink, 1983; Lucchini et al., 1984). Subsequently, studies have shown that GCN5 is a transcriptional associated histone acetyltransferase (Georgakopoulos and Thireos, 1992; Brownell et al., 1996). Its acetyltransferase activity seems to be dependent on the association in different multisubunit complexes, such as SAGA (Spt-Ada-Gcn5-Acetyltransferase), ADA (Ada2-Gcn5-Ada3), and SLIK/SALSA (SAGA-like) (Grant et al., 1999). Homologs of *S. cerevisiae* Gcn5 have been identified in many fungi, where they have been found to be involved in growth, development, regulation of secondary metabolism and virulence in many fungi (Canovas et al., 2014; Chang et al., 2015; Lan et al., 2016). In addition, in *A. nidulans* GcnE can increase acetylation levels of histone sites H3K9 and H3K14 during interaction with bacteria and secondary metabolites production is enhanced in the wake of the global increase of H3K14 acetylation (Nutzmann et al., 2011).

MYST proteins, the biggest family of HATs, are associated with a diverse variety of biological functions (Wang et al., 2013). In fungi, the most studied MYST histone acetyltransferases are ESA1 (essential Sas2-related acetyltransferase 1), SAS2 (Something About Silencing, also called KAT8), and SAS3 (related to SAS2). Sas2 is the catalytic subunit of the SAS HAT complex (Sas2p-Sas4p-Sas5p) and implicated in acetylation of H4K16 (Cavero et al., 2016). Besides, it plays important roles in the regulation of transcriptional silencing (Osada et al., 2001; Shia et al., 2005), DNA replication and cell cycle progression (Kimura et al., 2002; Zou and Bi, 2008). SAS3, as a catalytic subunit of the NuA3 complex (John et al., 2000), is the least studied MYST proteins in yeast. Simultaneous disruption of SAS3 and GCN5 results in an extensive loss of H3 acetylation and stagnation in the G2/M phase of the cell cycle (Howe et al., 2001). Recently, in *Metarhizium robertsii*, disruption of Hat1, a homolog of *S. cerevisiae* SAS3, increased the expression of orphan secondary metabolite genes and made a global loss of H3 acetylation (Fan et al., 2017).

Rtt109 is responsible for the acetylation of H3K56 (Schneider et al., 2006) More recently, Rtt109 was shown to function in maintaining genome stability and participate in DNA replication (Han et al., 2007), DNA damage repair (Chen et al., 2008) and to negatively regulate stress responsive genes in *S. cerevisiae* (Cheng et al., 2016).

*F. graminearum* is the major causal agent of Fusarium head blight (FHB) on various cereal crops (Bai and Shaner, 2004; Goswami and Kistler, 2004). It causes severe yield losses and the pathogen contaminates infested grains with mycotoxins, such as deoxynivalenol (DON) and its derivatives, which are serious threats to human and livestocks (Sutton, 1982). FHB disease is a global problem and incidence and severity increase in southwest and northern wheat and barley-growing regions in China (Yang et al., 2008; Zhang et al., 2012). Orthologs of yeast HAT genes are well conserved in filamentous ascomycetes and play an important role in cell regulation and metabolism including secondary metabolite production. However, to date, only one histone acetyltransferase ELP3 (Elongator protein 3) has been reported in *F. graminearum*, where it exerts pleiotropic effects on sexual development and virulence (Lee et al., 2014). Although ELP3 and GCN5 are both members of the GNAT family and each contains a N-acetyltransferase domain, they share lower protein sequence similarity. Recently, over-expression of histone acetyltransferase gene HAT1 in the gibberellin acid (GA)-deficient Δlae1 mutant of *Fusarium fujikuroi*, was shown to contribute to the expression of GA gene and production of GA (Niehaus et al., 2018). Since the function of HATs are of importance, it's imperative to find and research more HAT genes in Fusarium.

In this study, we investigated the functions of four candidate HATs in *F. graminearum*. Our data revealed that the four HATs were involved in hyphal development, conidiation, sexual reproduction, DON biosynthesis, stress responses, and pathogenicity in *F. graminearum* at varying degrees. Moreover, we showed that *FgSAS3* and *FgGCN5* are responsible for the acetylation of different lysine residues on histone H3. A microarray-based transcriptome analysis revealed differential expression of genes involved in secondary metabolism, sexual development, and virulence.

## Materials and methods

### Strains and culture conditions

The wild-type strain PH-1, deletion mutants and complementary strains of *F. graminearum* generated in this study were routinely cultured at 25°C on PDA (200 g potato, 20 g dextrose, 20 g agar, and 1 L ddH_2_O) agar plates and were preserved in 15% dimethyl sulfoxide (DMSO) at −80°C.

### Identification of histone acetyltransferase genes in *F. graminearum*

To identify putative histone acetyltransferase genes in *F. graminearum*, a BLASTp search with GCN5, RTT109, SAS2, and SAS3 of *S. cerevisiae, F. oxysporum, A. nidulans, N. crassa, S. pombe, and C. albicans* were performed in the *F. graminearum* genome database. The phylogenetic tree analysis was conducted using the neighbor-joining method in MEGA 5.0. Bootstrap analysis was conducted using 1,000 replicates in MEGA 5.0.

### Generation of gene deletion and complemented strains

To generate constructs for disruption of each of the four putative *F. graminearum* HATs gene, their flanking regions were amplified by PCR using wide-type PH-1 genomic DNA, extracted with the E.Z.N.A.® Fungal DNA Mini Kit (Omega Bio-tek, Inc. Orlando, USA), as the template. Primers for amplification of the upstream (primers UF+UR) and downstream (primers DF+DR) flanking regions are listed in (Table S2). Flanking sequences were ligated into plasmid pKH-KO (that carries the hygromycin resistance gene *HPH*). For the construction of gene complementation vectors, sequences, which include promoter, gene, and terminator regions are amplified by PCR using primers F+/R+ (Table S2), and were ligated into plasmid pKN (which carries the G418 resistance gene *NEO*) with the seamless assembly cloning kit (Clone Smarter Techology, USA). The gene deletion and complementation plasmids are digested and subsequent, transformed to PH-1 using the protocols described previously (Turgeon et al., 1987). Hygromycin B (AMRESCO USA) and geneticin (AMRESCO USA) were added to the final concentrations of 200 and 100 μg/mL, respectively, for transformant selection. Putative gene deletion and complementation mutants were identified by PCR assays with the relevant primers (Table S2), and were further validated by genome sequencing performed on the HiSeq X Ten sequencing system. Reads were aligned to reference genome by Burrows-Wheeler Aligner (Li and Durbin, 2009). Sam alignment files were converted to bam format, sorted, and indexed with samtools (Li and Durbin, 2009) and then visualized by Integrative Genomics Viewer (IGV) (Thorvaldsdóttir et al., 2013).

### Conidiation assays

For conidiation assays, one mycelial plugs (9 mm in diameter) of each strain, taken from the periphery of a 3-day-old colony, were inoculated in a 50-mL flask containing 30 mL of liquid carboxymethyl cellulose (CMC). Each strain was set up in three technical repetitions. The flasks were incubated at 25°C on a shaking table (180 rpm). After 4 days of cultivation, the liquid medium was filtered through three layers of sterile lens wiping paper, and the spores were resuspended in 25 mL sterile water. The number of conidia was counted for each strain using a haemocytometer. Conidial morphology and presence of septa were observed with a Leica TCS SP5 imaging system. The experiment was repeated three times independently. For growth assays, the growth rate on complete medium (CM), PDA and minimal medium (MM) plates were measured 3 days at 25°C after inoculation a 9 mm plug on the plates. To assay defects in responses to stress, final concentrations of 1 M NaCl, 1.2 M KCl, and 0.069% H_2_O_2_ were added to MM.

### Sexual reproduction assay

For sexual reproduction, mycelial plugs (9 mm in diameter) of each strain, taken from the edge of a 3-day-old colony, were placed onto carrot agar (400 g carrot, 20 g agar and 1 L ddH_2_O) medium and cultured at 25°C. After 4 days, aerial hyphae was stripped away and the incubation temperature was reduced to 23°C under a cycle of 12 h of UV-light and 12 h of darkness for 1 week. During which aerial hyphae were pressed down with 200 μL sterile 0.2% Tween-60 when they grow again. Observation of the perithecia was done using a digital microscope VHX-2000 (KEYENCE, USA). Triplicates were used for each strain and three independent repeats of the experiment were performed.

### DON production assays

Conidia of PH-1 and mutant strains were harvested from 4-day-old CMC cultures and resuspended at 10^6^ spores/mL. Of the spore suspension 500 μL was added to 50 ml of TBI medium in a 150-mL flask. TBI medium (Gardiner et al., 2009a,b) consists of 1 g KH_2_PO_4_, 0.5 g MgSO_4_·7H_2_O, 0.5 g KCl, 0.871 g L-arginine, 0.01g FeSO_4_·7H_2_O, 30 g sucrose, 200 μL of trace element solution (per 100 mL, 5 g citric acid, 5 g ZnSO_4_·6H_2_O, 50 mg NaMoO_4_·2H_2_O, 0.25 g CuSO_4_·5H_2_O, 50 mg MnSO_4_, 50 mg H_3_BO_3_), pH 4.5. In the case of Δ*FgGCN5* mycelial suspension was used for the inoculation of the TBI medium. The production of DON of each strain was determined based on three repetitive measurements. The flasks were incubated at 25°C in a shaker with 180 rpm. After 1 week of culture, the liquid medium was filtered with 0.22 μm syringe filter. Deoxynivalenol was measured using the DON Plate Kit ELISA from Beacon Analytical Systems (Portland, Maine, USA) according to the manufacturer's instructions. Briefly, 50 μL enzyme conjugates, 50 μL samples/standards, and 50 μL monoclonal antibody were added into corresponding test well in sequence. The microplate was shaken slightly and incubated for 10 min at room temperature. After that, the mixture in the microwells was drained off and the wells were washed 5 times with washing solution. Next, 100 μL of substrate solution was added to each well and the plate incubated at room temperature for 5 min. Finally, 100 μL of stop solution was added to each well and the absorbance at 450 nm wavelength was determined with a multimode reader (BioTek® Instruments, Synergy 4, USA). The concentration of each sample can be calculated according to the absorbance value. The experiment was repeated three times independently.

### Plant infection assays

Conidia were harvested from 5-day-old mungbean liquid medium (MBL) (Wei et al., 2017; 30 g mung bean, 1 L ddH_2_O, cook for 10 min, filtered and sterilized) and resuspended at 10^6^ conidia/mL in sterile water. For infection on flowering wheat (*cv*. Yangmai 158) grown in the greenhouse, heads were assessed as described previously (Seong et al., 2005; Ding et al., 2009). For each flowering wheat head, the spikelet at the lower-middle part was inoculated with 10 μL of conidial suspension. There were 30 replicates for each strain. The inoculated ear was sprinkled and put into a zip-lock bag to keep 100% humidity for 3 days. Two weeks after inoculation, the infected spikelets in each inoculated wheat head were recorded and pictures were taken. Because the Δ*FgGCN5* mutant was unable to form conidia, a mycelial suspension was used inoculum, and the same procedure was also performed on PH-1 as positive control.

Infection assays with tomato were done as previous described (Di Pietro et al., 2001). In short, a 10 μL aliquot of a conidial suspension was injected into wounded tomato after surface disinfection. For mutant Δ*FgGCN5*, again mycelial suspension was used as inoculum. There were three replicates for each strain. Inoculated tomatoes were incubated at 25°C and 100% humidity with 12 h of daylight in artificial climate incubator, and were photographed 3 days after inoculation.

The penetration behavior of each strain was also examined on cellophane membranes using a previously published protocol (Prados Rosales and Di Pietro, 2008). In brief, each strain was grown on MM covered with a layer of cellophane membrane on it. After 2 days of cultivation, the cellophane membrane with the colony was removed from each plate. Subsequently, the plates were incubated another day and mycelial growth on each plate was examined and photographed. All infection experiments described above were repeated three times independently.

### RNA extraction and qRT-PCR analysis

For total RNA isolation, mycelia of each strain were inoculated in TBI and cultured for 3 days at 25°C with 180 rpm in the dark. Mycelia were harvested by centrifugation and washed with sterilized water for three times. Excess water was then removed with filter paper and mycelia were ground in liquid nitrogen. Total RNA of each strain was extracted using the TaKaRa MiniBEST Plant RNA Extraction Kit (Takara Bio Inc., Dalian, China), and then used for reverse transcription to synthesize first-strand cDNA with TaKaRa PrimeScript™ RT reagent Kit with gDNA Eraser (Takara Bio Inc., Dalian, China). The expression of trichothecene biosynthesis related genes *FgTRI5, FgTRI6, FgTRI10*, and *FgTRI12* was determined by qRT-PCR and the expression of the TUB2 beta-tubulin gene was used as a endogenous reference (Jiang et al., 2016). The reactions were performed with the SYBR Premix Ex Taq II (Tli RNaseH Plus) (Takara Bio Inc., Dalian, China) and the 7500 real time PCR System (Applied Biosystems, Foster City, CA, USA). Primers are listed in supplementary information Table S2. The experiment was repeated three times independently. Fold expression was calculated based on expression in wide-type PH-1 by the 2^−ΔΔCt^ method (Livak and Schmittgen, 2001). For each gene, qRT-PCR data from three biological replicates were used to calculate the mean and standard deviation.

### RNA-seq analysis

For RNA-seq analysis, the Δ*FgSAS3*, Δ*FgGCN5*, and PH-1 strains were cultured in TBI medium for 3 days. Total RNA was isolated from the lyophilized mycelium as described above and mRNA was isolated using a Poly(A)Purist MAG kit (Ambion). DNA was removed by treatment with RNase-free DNAase (Qiagen), followed by column clean-up according to manufacturer's instructions, then Illumina TruSeq RNA Sample Preparation kits were used to make RNA-seq libraries. cDNA was sequenced with the HiSeq X Ten sequencing system. The RNA-seq reads (150 bp) were mapped to the genome of the *F. graminearum* strain PH-1 (http://fungi.ensembl.org/) using hisat2 (Kim et al., 2015). Sam-formatted alignment files were converted to bam format, sorted, and indexed with samtools (Li and Durbin, 2009). Identification of differentially expressed genes (DEGs) from RNA-seq data was conducted by using cufflinks (Trapnell et al., 2012). Detection of genes differentially enriched was analyzed with TBtools (https://github.com/CJ-Chen/TBtools). Primary metabolism-associated genes were download from The Fungal and Oomycete Genomics Resource Database (http://fungidb.org/) with GO term “0044238, primary metabolic process.” Secondary metabolism gene clusters were predicted by Sieber et al. (2014). Genes associated with sexual reproduction were from two previous reports (Hallen et al., 2007; Kim et al., 2015). Virulence-related genes were found from PHI-base database (http://www.phi-base.org/index.jsp).

### Western blot analysis

For western blot analyses, 500 μL of mycelium cultured in YEPD liquid medium were inoculated into 100 mL of TBI liquid medium and incubated at 25°C in a shaker with 180 rpm for 2 days. Mycelia were then harvested, washed with ddH_2_O and the excess water was absorbed by filter paper. Samples (0.2 g) of mycelium were ground in liquid nitrogen and homogenized with a vortex shaker in 1 mL lysis buffer composed of 50 mM Tris-HCl, pH 7.5, 100 mM NaCl, 5 mM ethylenediaminetetraacetic acid (EDTA), 1% Triton X-100, 2 mM phenylmethylsulphonylfluoride (PMSF) (P7626, Sigma-Aldrich) and 10 μL of protease inhibitor cocktail (P8215, Sigma-Aldrich). The homogenates were centrifuged at 15,000 rpm for 20 min at 4°C, and the supernatants were collected as total protein. The concentration of total protein was quantified by BCA protein assay kit (Solarbio, Beijing, China). Equal amounts of homogenate protein (50 μg) were subjected to 12.5% SDS-polyacrylamide gel electrophoresis (PAGE), electroblotted onto an immobilon-P polyvinylidene difluoride (PVDF) membrane (0.22 um, Millipore, Bedford, MA), and probed with primary antibodies H3 (Abcam Cat# ab1791, RRID:AB_302613), H3ac (Millipore Cat# 06-599, RRID:AB_2115283), H3K4ac (Cat# 39382, RRID:AB_2722568), H3K9ac (Cat# 39917, RRID:AB_2616593), H3K14ac (Cat# PTM-113, RRID:AB_2722570), H3K18ac (Cat# 39755, RRID:AB_2714186), H3K27ac (Cat# 39134, RRID:AB_2722569), and goat anti-rabbit IgG-HRP secondary antibody (Abcam Cat# ab6721, RRID:AB_955447). Protein bands were visualized by reaction with chemiluminescent HRP substrate (Millipore, Billerica, USA). The experiment was conducted three times independently. Images obtained by the three repeated experiments were processed by Adobe Photoshop CS6, and the sum of integral optical density (IOD SUM) of each protein in the PVDF membrane was measured after the background was dislodged by “Subtract Background” function using Image-Pro_Plus 6.0 software (Media Cybernetics, CA, United States). Finally, the mean IOD was calculated as a ratio of IOD SUM relative to area.

### Statistical analysis

All data were shown as mean ± standard error (SE) and all analyses were conducted with using the Statistical Analysis System (Cary, NC, USA, version 9.2). The differences between two groups were subjected to the independent sample *t*-test (*P* < 0.05). The differences among different groups were subjected to a least significant difference (LSD) test at the *P* < 0.05. ^*^ and ^**^ indicate significant differences at *P* < 0.05 and *P* < 0.01, respectively, compared with PH-1.

## Results

### *In silico* analysis of four HATs in *F. graminearum*

Putative *F. graminearum* GCN5 (*FgGCN5*; *FGRRES_00280_M*), RTT109 (*FgRTT109*; *FGRRES_13497*), SAS2 (*FgSAS2*; *FGRRES_06047*), SAS3 (*FgSAS3*; *FGRRES_08481*) orthologs were identified from the genome data-base (King et al., 2015) through amino acid sequence homology searches with the BLASTP algorithm using GCN5, RTT109, SAS2, and SAS3 of *S. cerevisiae, F. oxysporum, A. nidulans, N. crassa, S. pombe, and C. albicans as* queries. The amino acid sequence similarities of the *F. graminearum* sequences with the orthologs in the six species are listed in Table S1. These putative HATs of *F. graminearum* had the highest similarity with *F. oxysporum*, followed by *N. crassa* and *A. nidulans*. *FgGCN5* was ≥ 57% similar to the corresponding six species proteins, suggesting that GCN5 is relatively conserved in these species. While for the other three HATs, the species vary widely. The phylogenetic tree analysis revealed that *F. graminearum* HATs are closely related to putative HATs orthologs from *F. oxysporum* (Figure S1).

### Generation of four HAT genes deletion and complementation strains in *F. graminearum*

**Figure 1:**
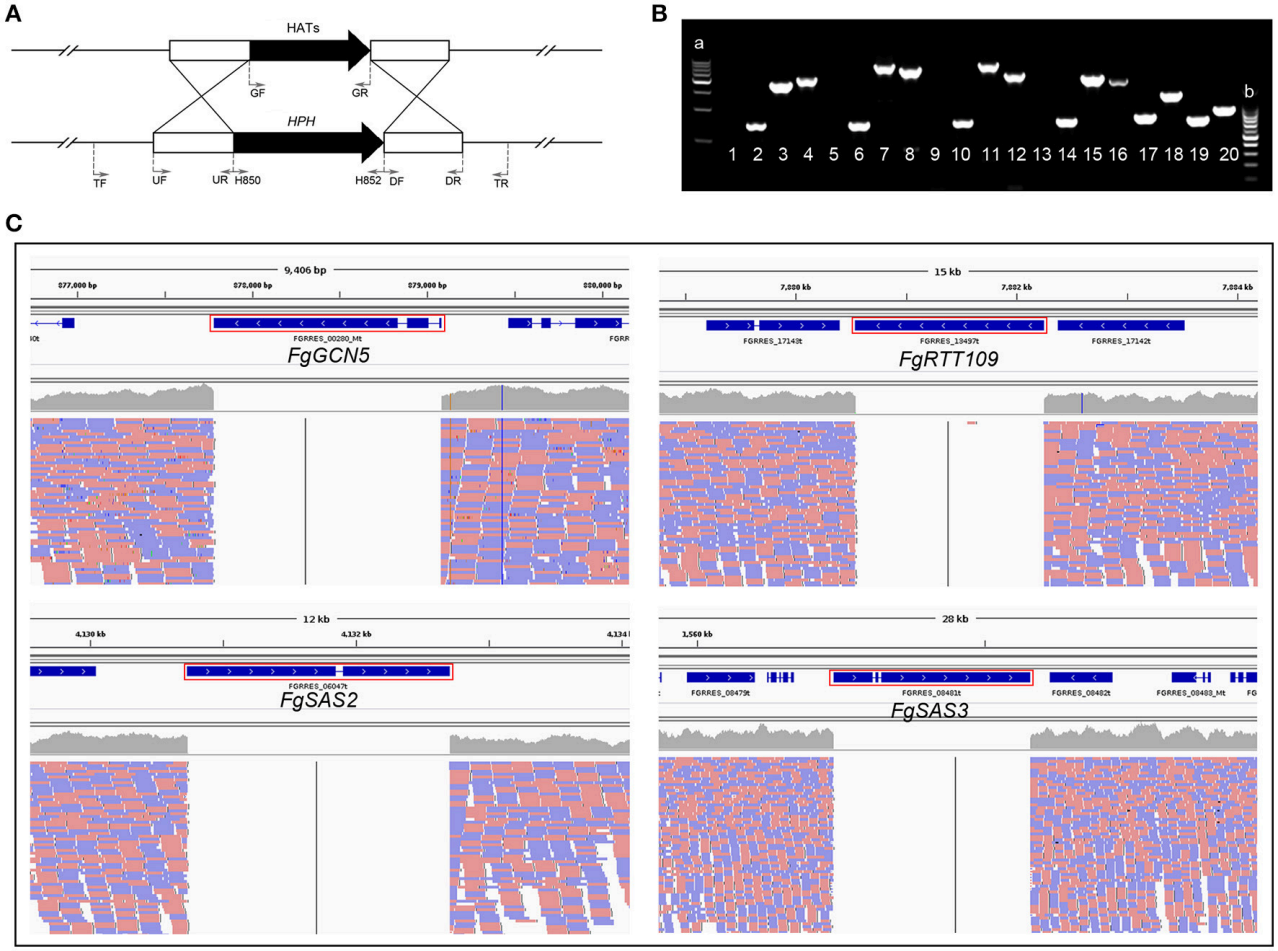
Deletion of four HATs from the genome of wild-type strain PH-1, respectively. **(A)** Schematic representation gene disruption strategy. HAT gene and the hygromycin resistance cassette (HPH) are denoted by black and arrows. The annealing sites of primers UF/UR, DF/DR, TF/TR, GF/GR, H850/H852 are indicated by small black arrows. **(B)** Electropherogram of PCR products. a: 500 bp DNA ladder maker; b: 100 bp DNA ladder maker; Lane 1, 5, 9, 13: PCR products amplified with primers GF/GR using Δ*FgRTT109*, Δ*FgSAS2*, Δ*FgSAS3* and Δ*FgGCN5* as a template, respectively; Lane 2, 6, 10, 14: PCR products amplified with primers H850/H852 using Δ*FgRTT109*, Δ*FgSAS2*, Δ*FgSAS3*, and Δ*FgGCN5* as a template, respectively. Lane 3, 7, 11, 15: PCR products amplified with primers TF/H852 using Δ*FgRTT109*, Δ*FgSAS2*, Δ*FgSAS3*, and Δ*FgGCN5* as a template, respectively. Lane 4, 8, 12, 16: PCR products amplified with primers H850/TR using Δ*FgRTT109*, Δ*FgSAS2*, Δ*FgSAS3*, and Δ*FgGCN5* as a template, respectively. Lanes 17-20: PCR products amplified with primers GF/GR using FgRTT109c, FgSAS2c, FgSAS3c, and PH-1 as a template, respectively. **(C)** Genome re-sequencing of four mutants. The location of *FgGCN5, FgRTT109, FgSAS2, FgSAS3*, and on chromosomes was marked in red box.

For a detailed functional analysis of the four HATs, we generated deletion mutants by replacing the genes of *FgGCN5, FgRTT109, FgSAS2*, and *FgSAS3* with the hygromycin-resistance gene as a selectable marker in *F. graminearum* wild-type strain PH-1, respectively following the strategy shown in Figure 1A. Deletion mutants were identified by PCR analysis with three primer pairs TF/H852, H850/H852, H850/TR (Figure 1B; Table S2). Corresponding mutants, Δ*FgGCN5*, Δ*FgRTT109*, Δ*FgSAS2*, and Δ*FgSAS3*, used in all the following experiments were also confirmed by whole genome resequencing (Figure 1C). The complemented strains, FgRTT109c, FgSAS2c, and FgSAS3c were constructed by ectopic insertion of the target gene into the genome of the corresponding mutant strains, and were identified by geneticin-resistance and PCR on the geneticin gene (primers GF/GR; Table S2). As the Δ*FgGCN5* mutant did not produce conidia and grew extremely slowly, we tried to use young mycelia grown on different media to obtain protoplasts required for the transformation. Unfortunately, all transformation efforts were unsuccessful and no complementation strain was obtained for the Δ*FgGCN5* mutant.

### Involvement of HATs in the regulation of hyphal growth and stress response

**Figure 2:**
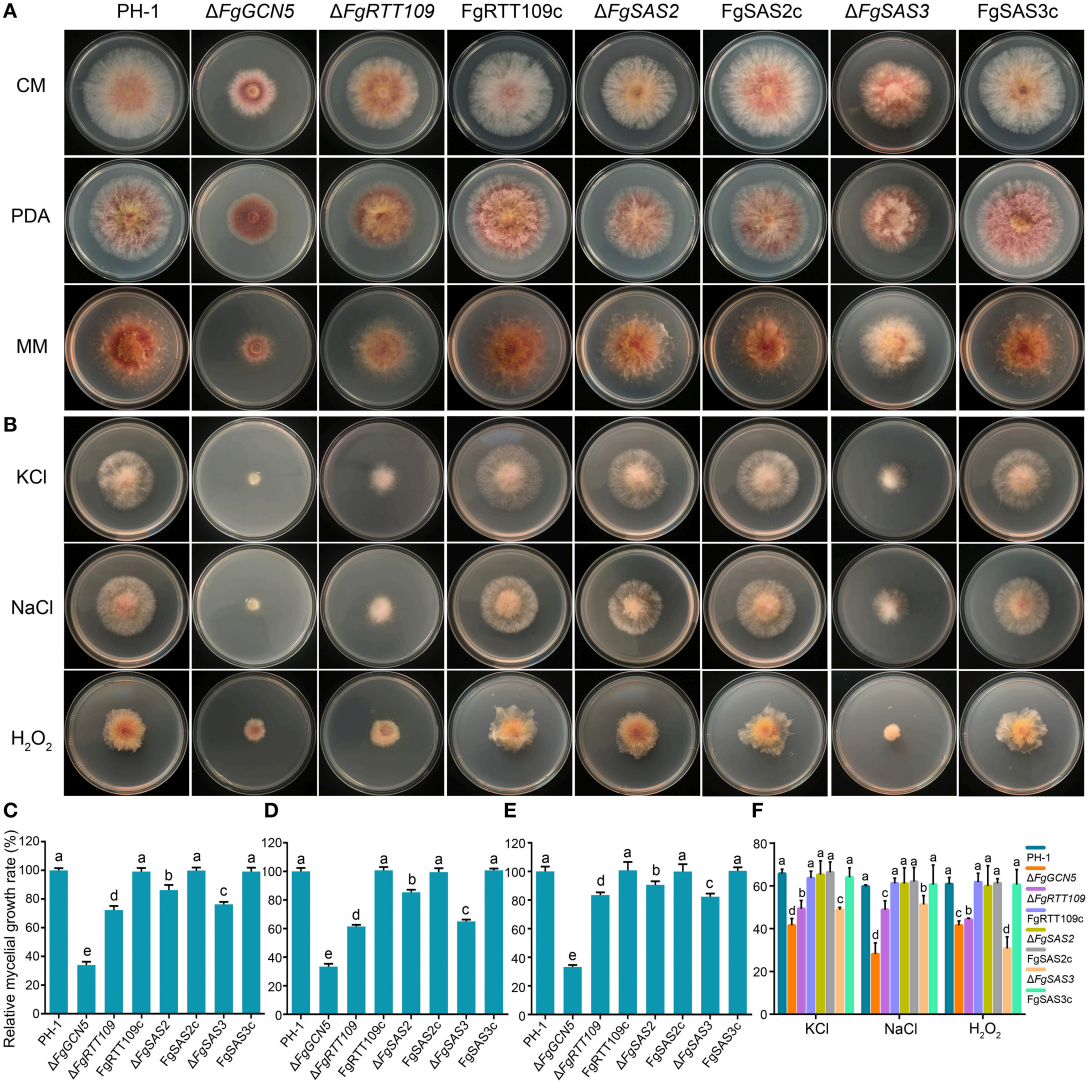
Impacts of four HATs deletion strains on *F. graminearum* mycelial growth and their sensitivity to osmotic and oxidative stress. **(A)** The wild-type PH-1, deletion mutants (Δ*FgGCN5*, Δ*FgRTT109*, Δ*FgSAS2*, and Δ*FgSAS3*) and the complemented strains (FgRTT109c, FgSAS2c, and FgSAS3c) were grown on PDA, CM, and MM at 25°C. **(B)** Sensitivity of each strain to osmotic stress and oxidative stress. Comparisons were made on MM amended with 1 M NaCl or 1.2 M KCl or 0.069% H_2_O_2_. **(C,D,E)** Relative mycelial growth rate was determined after incubation for 3 days on PDA, CM, and MM. Bars denote standard errors from five repeated experiments. Values on the bars followed by the same letter are not significantly different at *P* = 0.05. **(F)** Mycelial growth inhibition was examined after each strain was incubated for 3 days on MM amended with 1 M NaCl or 1.2 M KCl or 0.069% H_2_O_2_. Bars denote standard errors from five repeated experiments. Values on the bars followed by the same letter are not significantly different at *P* = 0.05.

The colony morphology was compared of each mutant following growth on three different solid media: PDA, CM, and MM. The growth trend we observed of the same mutant strain on these different media was consistent (Figure 2A). Δ*FgSAS3* and Δ*FgGCN5* displayed different growth phenotypes on the three culture media compared with PH-1. For Δ*FgSAS3*, pigment production reduced significantly on MM and compact colonies formation with crisped, short aerial hyphae on the three different media (Figure 2A). In contrast, pigment production by Δ*FgGCN5* was significantly increased on PDA and aerial hyphae were sparse on all three tested media (Figure 2A). Additionally, The growth rate of mutants Δ*FgGCN5*, Δ*FgRTT109*, Δ*FgSAS2*, and Δ*FgSAS3* was reduced by 70, 40, 20, and 40%, respectively, compared with wild-type progenitor on these three media (*P* < 0.05; Figures 2C–E). The growth defects of Δ*FgRTT109*,Δ*FgSAS2*, and Δ*FgSAS3* mutants on solid media were restored by genetic complementation with the wild-type HAT genes in the corresponding strains. These results indicate that all four HATs play important roles in the regulation of hyphal growth in *F. graminearum*.

To explore the roles of *FgGCN5, FgRTT109, FgSAS2*, and *FgSAS3* in response to different kinds of stress, the four mutant strains and the corresponding complementation strains were incubated on MM supplemented with osmotic stress agents (1 M NaCl, 1.2 M KCl) or an oxidative stress agent (0.069% H_2_O_2_). Mutant Δ*FgSAS2* did not show any response to these stress conditions as shown in Figures 2B,F. In contrast, Δ*FgGCN5*, Δ*FgRTT109*, and Δ*FgSAS3* exhibited significant growth reduction in response to these three stressors (*P* < 0.05). In the complemented strains FgRTT109c and FgSAS3c these stress responses were restored to the wild-type level (Figures 2B,F).

### *FgGCN5, FgRTT109*, and *FgSAS3* are required for sexual and asexual reproduction

**Figure 3:**
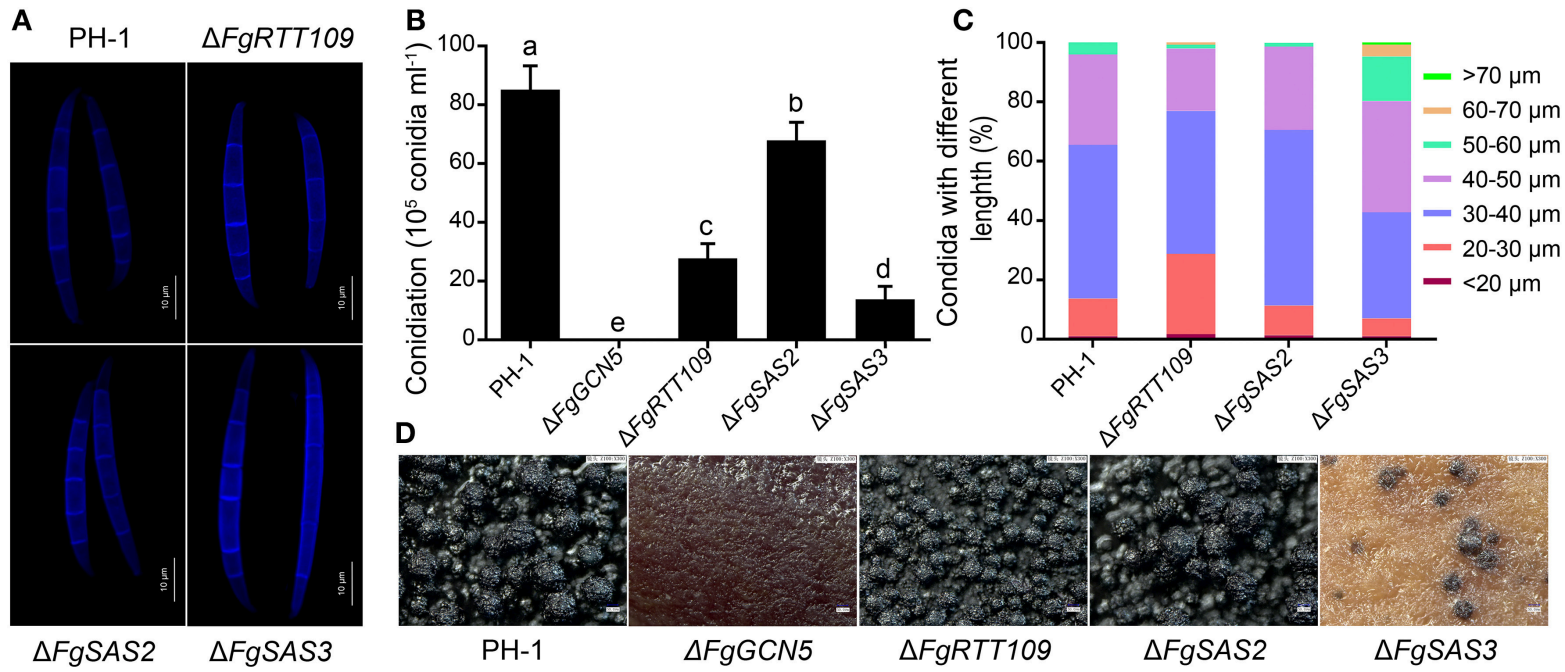
Morphologies of conidia and the formation of perithecia. **(A)** Microscopic observations of conidial morphology of PH-1, Δ*FgRTT109*, Δ*FgSAS2*, and Δ*FgSAS3*. Differential interference contrast (DIC) images of conidia stained with calcofluor white were captured with an electronic microscope. Scale bar = 10 μm. **(B)** The number of conidia produced by PH-1, Δ*FgRTT109*, Δ*FgSAS2* and Δ*FgSAS3* strains. Error bars indicate the standard deviations of three repeated experiments. Values on the bars followed by the same letter are not significantly different at *P* = 0.05. **(C)** Percentages of conidia with different lengths in PH-1, Δ*FgRTT109*, Δ*FgSAS2* and Δ*FgSAS3* strains. **(D)** Microscopic observations of perithecia. Perithecia formation of PH-1, Δ*FgGCN5*, Δ*FgRTT109*, Δ*FgSAS2*, and Δ*FgSAS3* were photographed by digital microscope VHX-2000 at 2 weeks post-fertilization after sexual induction on carrot agar. Mutant Δ*FgGCN5* failed to produce perithecia.

To determine effects of the four HATs deletion mutants on conidiation, fresh mycelial plugs of each strain were inoculated in CMC medium. After four days of incubation in a shaker at 180 rpm not detectable changes in conidial morphology were observed either in Δ*FgRTT109* or Δ*FgSAS2* (Figure 3A). Twenty percentage conidia of Δ*FgSAS3*, however, were longer than those of PH-1 (Figures 3A,C). Δ*FgSAS2* exhibited a slight reduction in conidiation (Figure 3B). However, Δ*FgRTT109* and Δ*FgSAS3* showed dramatically reduced conidiation in comparison with the wild-type PH-1 (Figure 3B). In Δ*FgGCN5* the ability of asexual reproduction was totally lost. These results showed that two HATs, *FgSAS3* and *FgGCN5* play vital roles in the modulation of production and morphology of conidia.

Ascospore formation and discharge are regarded as the primary infection source for FHB epidemics in the spring (Goswami and Kistler, 2004). Moreover, perithecia and perithecia-associated hyphae that are left on plant debris provide the primary ways for survival during winter. When self-fertilized, the *FgSAS3* deletion mutant produced few perithecia, while Δ*FgGCN5* failed to produce any perithecia after up to 2 weeks of induction compared with the wild-type PH-1 and complemented strains (Figure 3D). Mutant Δ*FgRTT109* produces perithecia that were smaller than those of wide type PH-1 and complementation strains. Upon squashing the perithecia only barely ascospores were released from mutant Δ*FgRTT109*. Perithecia with similar morphology to those of PH-1 were observed in Δ*FgSAS2* mutants (Figure 3D). These results suggested that *FgRTT109, FgSAS3, and FgGCN5* are essential for female fertility.

### *FgSAS3* and *FgGCN5* are required for plant infection and DON biosynthesis

**Figure 4:**
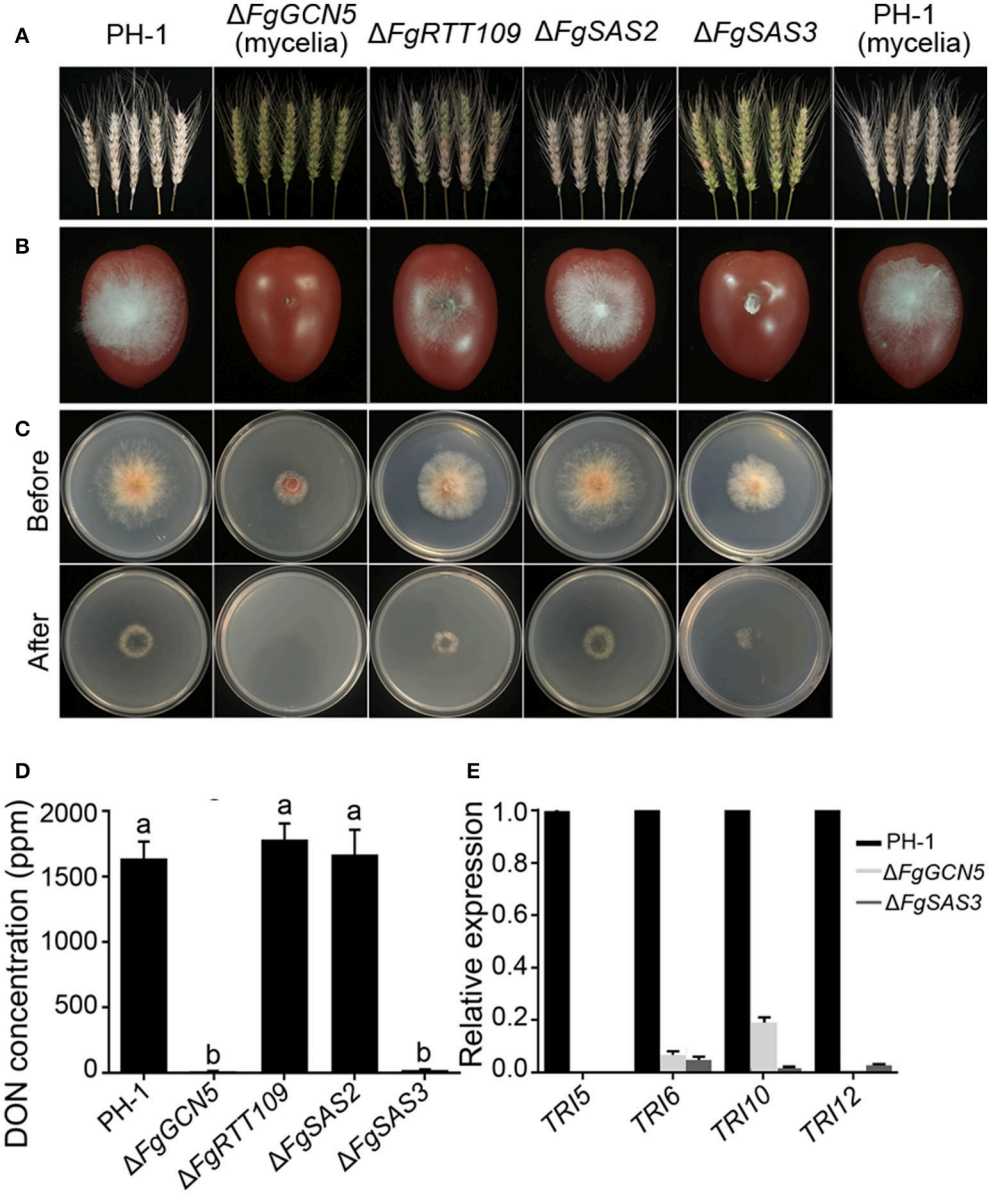
Comparison of virulence, DON production and the relative expression levels of selected trichothecene genes in PH-1 and the deletion mutants. **(A)** Pathogenicity on flowering wheat heads. Flowering wheat heads were point inoculated with a conidial suspension at 10^6^ conidia/mL of the wild type strain PH-1, Δ*FgRTT109*, Δ*FgSAS2*, and Δ*FgSAS3*. In the case of Δ*FgGCN5*, a mycelial suspension was used to inoculate wheat ears. Infected wheat heads were photographed 2 weeks after inoculation. **(B)** Pathogenicity on cherry tomato fruits. Cherry tomatoes were inoculated with a conidial suspension at 10^6^ conidia/mL of PH-1, Δ*FgRTT109*, Δ*FgSAS2*, and Δ*FgSAS3*. A mycelial suspension of Δ*FgGCN5* was inoculated into cherry tomatoes. Fruits were photographed 3 days after inoculation. **(C)** Penetration of PH-1 and four deletion mutants through cellophane membrane. Fungal colonies were grown for 2 d at 25°C on top of a cellophane membrane placed on minimal medium (MM) and were photographed (Before). The cellophane membranes with the fungal colonies were removed, and the plates were incubated an additional 24 h to examine the presence of mycelial growth on the plates (After). **(D)** DON production byΔ*FgGCN5*, Δ*FgRTT109*, Δ*FgSAS2*, and Δ*FgSAS3* mutants compared with the wild type. The amounts of DON produced by each strain were determined after 7 days of inoculation into TBI liquid medium. Line bars in each column denote standard errors of three replicated experiments. Values on the bars followed by the same letter are not significantly different at *P* = 0.05. **(E)** Relative expression levels of *TRI5, TRI6, TRI10*, and *TRI12* in the wild-type PH-1, Δ*FgSAS3* and Δ*FgGCN5* assayed by qRT-PCR. The relative expression levels of TRI genes in Δ*FgSAS3* and Δ*FgGCN5* are the relative amounts of cDNA of the gene in the wild-type progenitor. Line bars in each column denote standard errors of three repeated experiments.

Infection assays of the HATs mutants were performed by inoculation of flowering wheat heads and cherry tomato fruits. The wild-type strain caused scab symptoms in nearly 100% of spikelets of inoculated wheat heads and formed dense mycelium on inoculated tomato fruits. Δ*FgSAS2* mutant demonstrated equivalent symptoms as PH-1. The Δ*FgRTT109* mutant was significantly reduced in virulence and caused symptoms on 50% of the spikelets, while hyphae grew sparsely on cherry tomato fruits; significantly less severe symptoms than PH-1. However, the Δ*FgSAS3* and Δ*FgGCN5* mutants only showed symptoms on the inoculated wheat kernels and spread to neighboring spikelets on the same head was never observed (Figure 4A). In addition, no apparent infection of cherry tomato fruits was observed with these mutants (Figure 4B). For further analysis of the virulence defects, the penetration behavior of these mutants was also examined on cellophane membranes. As shown in Figure 4C, except for Δ*FgSAS2*, Δ*FgRTT109* and Δ*FgSAS3* were greatly reduced in their penetration capacity of cellophane sheet, while Δ*FgGCN5* was unable to penetrate cellophane sheet altogether. These results indicate that Δ*FgRTT109*, Δ*FgSAS3*, and Δ*FgGCN5* are essential for the infection of host plant.

*F. graminearum* produces various mycotoxins during interaction with plants, and the mycotoxin deoxynivalenol (DON) is a virulence factor that helps the fungus colonization and spread within spikes (Proctor et al., 1995; Jansen et al., 2005; Seong et al., 2009). Thus, *in vitro* deoxynivalenol production by the four deletion mutants was also examined. We determined DON production in 7-day-old mycelium after germination of 5 × 10^5^ conidia in TBI liquid culture. The DON production levels of the Δ*FgSAS3* and Δ*FgGCN5* mutants were almost zero compared to that of the wild-type strain or the Δ*FgRTT109*, Δ*FgSAS2* mutants (Figure 4D). To further confirm these results, we analyzed the expression levels of four trichothecene biosynthesis genes *Tri5, Tri6, Tri10*, and *Tri12* by quantitative real-time RT-PCR using RNA samples isolated from mycelia grown in TBI for 3 days. The expression levels of four TRI genes in Δ*FgSAS3* and Δ*FgGCN5* were sharply down-regulated compared with those in wild-type (Figure 4E). These results were consistent with the profiles of DON production in Δ*FgSAS3*, Δ*FgGCN5*, and wild-type progenitor, which indicates that *FgSAS3* and *FgGCN5* play an important role in the regulation of deoxynivalenol biosynthesis in *F. graminearum*.

### Histone acetylation targets of *FgSAS3* and *FgGCN5*

**Figure 5:**
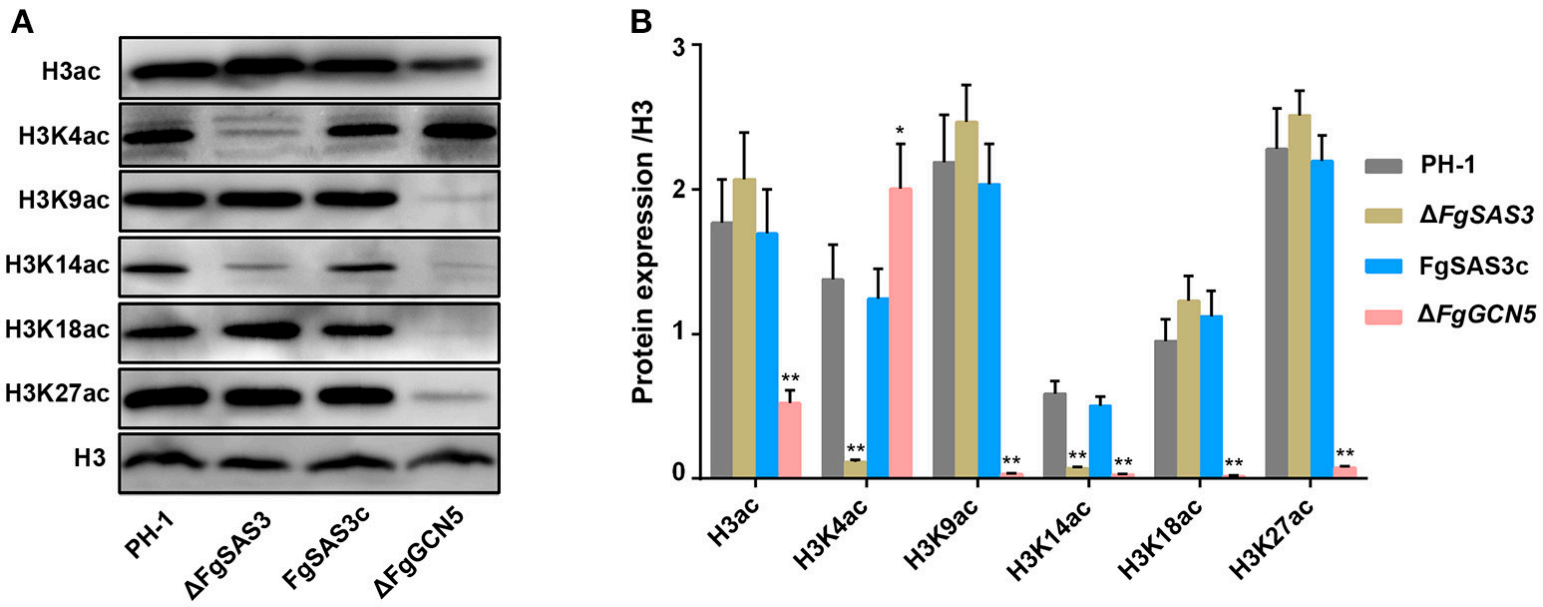
Western blotting analysis of proteins that extracted from PH-1, Δ*FgSAS3*, FgSAS3c, and Δ*FgGCN5*, respectively. **(A)** The respective strains were grown for 3 days in liquid TBI medium. The antibody anti-acetyl histone 3 (H3ac), anti-acetyl H3K4 (H3K4ac), anti-acetyl H3K9 (H3K9ac), anti-acetyl H3K14 (H3K14ac), anti-acetyl H3K18 (H3K18ac), and anti-acetyl H3K927(H3K27ac) were used for detection of alterations of acetylation levels. Antibody H3 was used as a loading reference. **(B)** Quantification of western blot signals in the three replicates. Data were expressed as mean IOD (relative to H3) ± standard error. *P < 0.05, **P < 0.01.

To test whether histone H3 acetylation levels were altered in the Δ*FgSAS3* and Δ*FgGCN5* mutants, we carried out western blot analyses with specific antibodies, directed against H3ac (acetylated H3 at the N-terminus), H3K4ac, H3K9ac, H3K14ac, H3K18ac, and H3K27ac, while antibody against H3 was used as a loading control. As shown in Figure 5, the levels of H3K4ac and H3K14ac were significantly decreased (*P* < 0.01) in the Δ*FgSAS3* mutant compared to the WT, while intensities of signals for H3ac, H3K9ac, H3K18ac, and H3K27ac were equivalent with the WT. In the Δ*FgGCN5* mutant, the detected signals for H3ac, H3K9ac, H3K14ac, H3K18ac, and H3K27ac were strongly decreased. In fact, the acetylation level of H3K4 in the Δ*FgGCN5* was significantly higher (*P* < 0.05) than that in PH-1 (Figure 5B). No reduction of the signal for histone H3 was observed in any of the mutants, suggesting that all reductions in H3 acetylation were lysine-specific (Figure 5A). The decreased levels of histone acetylation in the *FgSAS3* deletion mutant were completely recovered in gene complementation strain FgSAS3c. These results indicate that *FgSAS3* specially catalyzes acetylation of H3K4 and H3K14, whereas *FgGCN5* is responsible for lysine acetylation at residues 9, 14, 18, and 27.

### Identification of differentially expressed genes (DEGs) from Δ*FgSAS3* and Δ*FgGCN5* mutants

Since deletion of *FgSAS3* or *FgGCN5* has an appreciably impact on the virulence and the production of DON of *F. graminearum*, a genome-wide microarray analysis was performed using total RNA extracted from mycelia of Δ*FgSAS3*, Δ*FgGCN5* mutants as well as the wide type PH-1 cultured in TBI medium for 3 days. DEGs were selected from a fold change (Log2 fold change >=1 or <=-1). Analysis of the transcriptional profiles revealed a total of 2438 DEGs (1097 up-regulated and 1281 down-regulated) and 3375 DEGs (1296 up-regulated and 2079 down-regulated) in Δ*FgSAS3* and Δ*FgGCN5*, respectively. Among these DEGs, 767 genes were up-regulated and 948 were down-regulated in both mutants (Table S3, Figure S2). Notably, in Δ*FgGCN5* mutant, the number of genes down-regulated is much higher than that of up-regulated, suggesting *FgGCN5* mainly has positive regulatory effects on gene expression. The GO enrichment analysis revealed that several categories of biological processes were enriched among the DEGs of Δ*FgGCN5* mutant, including ribosome biogenesis, rRNA processing, ncRNA processing, and cellular amino acid metabolic process, while DEGs from the Δ*FgSAS3* mutant are mainly responsible for oxidation-reduction, transmembrane transport and carbon metabolism (Table S4).

### Transcription factors selected from the DEGs

Transcription factors (TFs) are essential players in the link between signal flow and target gene expression (Shelest, 2008). The entire repertoire of TFs in *F. graminearum* has been identified and their phenotypes have been systematically analyzed (Son et al., 2011). In our analyses, 108 (38 up-regulated and 70 down-regulated) and 181 (73 up-regulated and 108 down-regulated) TFs were identified among the DEGs in the Δ*FgSAS3* and Δ*FgGCN5* mutant, respectively (Table S5). In the Δ*FgGCN5* mutant, TFs with a specific and essential function for mycelia growth (19 TFs), conidia production (7 TFs), sexual development (18 TFs), virulence (17 TFs), and DON biosynthesis (11 TFs), were down-regulated in transcriptional profiles. In the Δ*FgSAS3* mutant, there are 15, 7, 14, 10, and 14 TFs, respectively, related to the above developmental processes and almost all of them are down-regulated (Table S5).

### *FgSAS3* and *FgGCN5* have an important regulatory role in secondary metabolism

**Figure 6:**
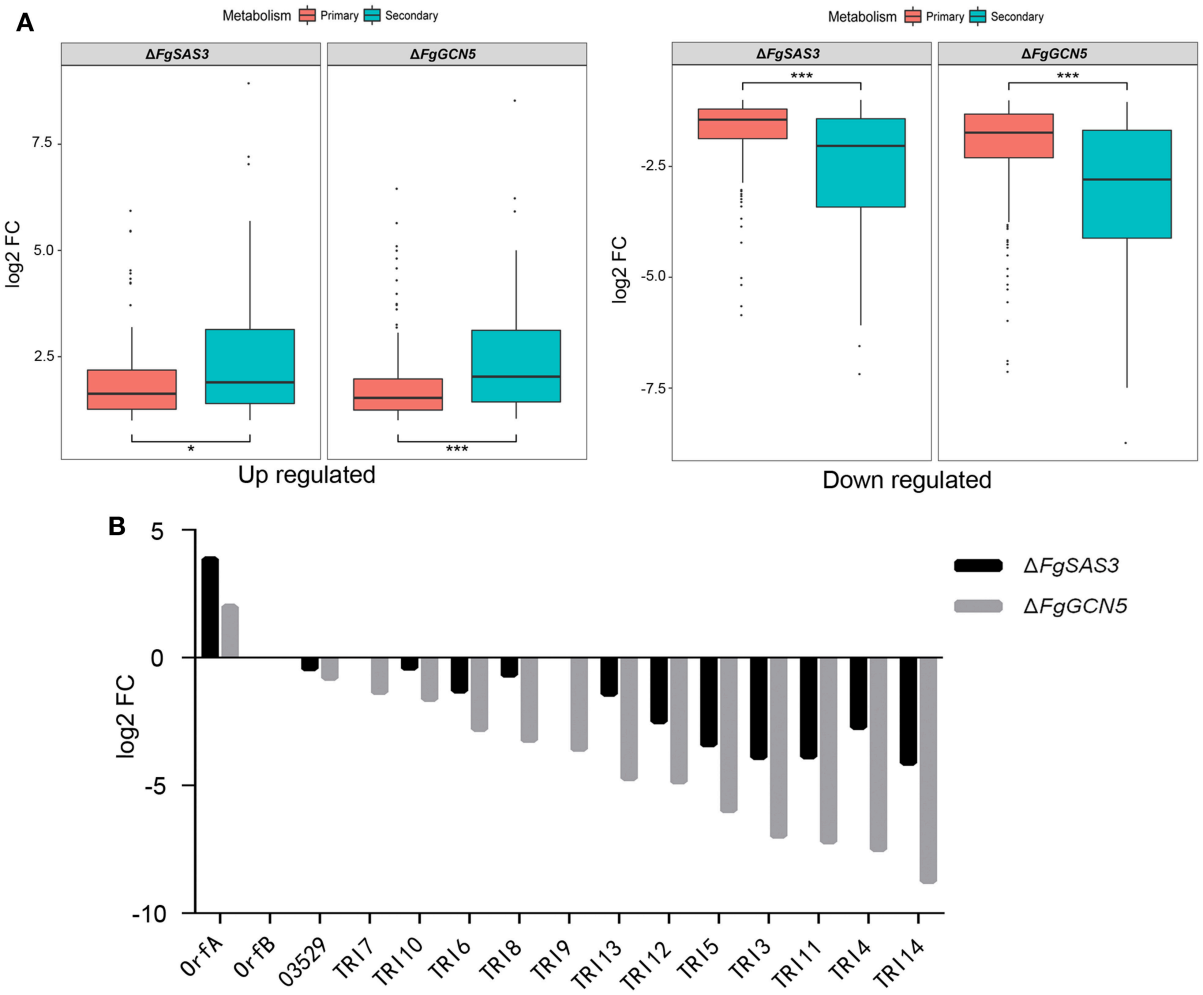
DEGs in primary and secondary metabolism and expression of all trichothecene biosynthesis related genes. **(A)** Boxplots for distribution of DEGs in primary and secondary metabolism. Primary metabolic genes were affected to a lesser extent compared to secondary metabolic genes in both mutants. Mild outliers were indicated by dot and asterisks highlight extreme outliers. *P < 0.05, ***P < 0.001. **(B)** Expression of all trichothecene biosynthesis related genes from transcriptome analyses. Almost all of the genes were down regulated in Δ*FgSAS3* and Δ*FgGCN5* mutants.

It is reported that histone acetyltransferases play a pivotal role in the activation of the secondary metabolite (SM) cluster in *A. nidulans* (Soukup et al., 2012). To elucidate the influence of *FgSAS3* and *FgGCN5* on the expression of primary metabolites (PMs) and 67 tentative SM gene clusters recognized in *F. graminearum* (Sieber et al., 2014), we identified DEGs within these metabolite gene clusters. In total, 23.2 and 28.5% SM genes of Δ*FgSAS3* and Δ*FgGCN5* were expressed significantly different, which exceeded the ratio of DEGs in PM genes (15.8 and 25.1%). In addition to more differently expressed SM genes, the expression ratios SM DEGs were also significant higher than PM DEGs (*t*-test, *P* < 0.05; Figure 6). This result indicated that secondary metabolism was significantly more affected than primary metabolism by disruption of *FgSAS3* and *FgGCN5*. In total, 165 genes belonging to 55 SM clusters were identified in the DEGs from the Δ*FgSAS3* mutant, and 202 genes belonging to 62 SM clusters were identified in the DEGs from the Δ*FgGCN5* mutant (Table S6). In both knock-out mutants, all genes in five clusters (C02, C23, C28, C49, C62) are downregulated, with the metabolites of C23, C28 and C49 being respectively trichothecene (Figure 6B), carotenoid and butenolide (Harris et al., 2007). All DEGs genes in four gene clusters (C21, C44, C53, C61) are upregulated, and metabolites of C21 and C53 are triacetylfusarinine and a precursor of the insoluble perithecial pigment, respectively. Furthermore, DEGs in cluster C13, whose metabolic product is aurofusarin (golden yellow to red/purple pigments), are downregulated in Δ*FgSAS3* and upregulated in the Δ*FgGCN5* mutant. In the Δ*FgSAS3* mutant, 18 DEGs that encoded signature enzymes (key enzymes in SM clusters) were in 17 SM clusters. Among these, a key enzyme (FGRRES_03537, *TRI5*) that participates in trichothecene biosynthesis was downregulated. Members of three polyketide synthase (PKS) gene clusters were upregulated: PKS22, PKS24, and PKS28, which is responsible for the biosynthesis of zearalenone, fusarielin, and orcinol/orsellinic acid, respectively. Three non-ribosomal peptide synthetase genes (NRPS6, NRPS8, NRPS18) were also identified. NRPS6 is involved in triacetylfusarinin metabolism, while metabolites of the other two NRPS are unknown. Moreover, we identified 27 genes that encoded tailoring enzymes and 37 transporters (Table S6). In the DEGs from the Δ*FgGCN5* mutant, 16 genes that encode signature enzymes were distributed across 15 SM clusters. Similarly, *TRI5* was downregulated dramatically. Five signature enzymes that belong to PKS13 (unknown metabolites), PKS22 (participate in the biosynthesis of zearalenone), PKS23 (unknown metabolites), PKS26 (involved in aurofusarin metabolism), and PKS28 (orcinol/orsellinic acid), respectively, are all upregulated. NRPS6 and NRPS8 were also identified and they were up-regulated and down-regulated respectively. Besides, we identified 31 genes that encode tailoring enzymes and 32 transporters (Table S6).

### *FgSAS3* and *FgGCN5* affect the expression of sexual reproduction related genes

It was reported that 37 genes are involved in the sexual development of *F. graminearum* (Kim et al., 2015). Among these genes, 10 DEGs were identified in the two mutants (Table S7). Five downregulated genes in both mutants, are responsible for perithecium formation, among which three of these genes being TFs (FGRRES_00404, FGRRES_15782, FGRRES_16753) and the other two being O-methylase (FGRRES_13708) and TF carrying-homeodomain (FGRRES_09019) respectively (Kim et al., 2015). While the upregulated genes have lighter effect on sexual reproduction, such as perithecia maturation or cirrhi formation (Kim et al., 2015). In addition to sexual development-specific functions, they are involved in a variety of metabolic pathways (FGRRES_06059, FGRRES_09896, FGRRES_10825, FGRRES_13708), transcriptional regulation (FGRRES_00404, FGRRES_09019, FGRRES_16753), RNA inference (FGRRES_00348), perithecial pigment (FGRRES_17168) (Gaffoor et al., 2005), cytoskeleton dynamics (FGRRES_15782) and chromatin silencing (FGRRES_13162). Hallen and co-workers identified 2086 probe sets that are particularly expressed during sexual development on carrot agar (Hallen et al., 2007), which implies these genes play important roles in sexual reproduction. Within these probe sets, 91 and 86 DEGs were identified in Δ*FgSAS3* and Δ*FgGCN5* mutant, respectively (Table S7), although hyphae were cultured in TBI medium and not on carrot agar. These results revealed that *FgSAS3* and *FgGCN5* may affect sexual reproduction through regulating the expression of the above genes.

### *FgSAS3* and *FgGCN5* are involved in various metabolic processes affecting pathogenicity of *F. graminearum*

In order to find out how both mutant strains affected the expression of virulence-related genes, we downloaded the pathogen-host interactions database of *F. graminearum* from PHI-base (http://www.phi-base.org/index.jsp), and screened for all the genes that putatively affect virulence (Table S8). In total, 41 DEGs (32 genes down- and 9 genes up-regulated) and 66 DEGs (54 down- and 12 up-regulated genes) were identified from the Δ*FgSAS3* and Δ*FgGCN5* mutants, respectively. Of these 36 (28 down- and 8 up-regulated) are present in both mutants. Virulence-associated DEGs from these two mutants include transcription factor, protein kinase, ABC transporter, regulators of G-protein, NRPSs, and trichothecene biosynthesis genes (Table S9), which suggest *FgSAS3* and *FgGCN5* are involved in numerous processes of metabolism to affect pathogenicity of *F. graminearum*.

## Discussion

Histone acetylation often favors the formation of euchromatin and strongly affects nuclear activities. It also regulates multiple cellular processes, including survival, cell proliferation, differentiation, and motility in a variety of organisms (Grunstein, 1997; Struhl, 1998; Brosch et al., 2008; Choudhary et al., 2014). Therefore, characterizing the function of HATs is an important way to illuminate the critical roles of these epigenetic processes. In this study, we identified four HATs genes encoding orthologs of yeast HATs in *F. graminearum*, which are named *FgGCN5, FgRTT109, FgSAS2*, and *FgSAS3*, respectively. To elucidate the functions of these HATs in *F. graminearum*, we created deletion mutants and analyzed their functions in growth, asexual and sexual reproduction, stress responses and virulence. We found that both *FgSAS3* and *FgGCN5* were more important for vegetative growth, conidiation, sexual reproduction, DON production, virulence than the other two genes. So further studies were conducted to determine the role of *FgSAS3* and *FgGCN5* in the regulation of expression of different genes and acetylation of H3 in *F. graminearum*.

In histone H3, the sites of acetylation include at least 5 highly conserved lysine residues, designated as K9, K14, K18, K23, and K27 (Kurdistani et al., 2004; Pham et al., 2007; Xue-Franzen et al., 2013). Since methylation and acetylation of the same lysine residues are mutually exclusive, the function of H3K4 acetylation has received little attention. However, in addition to being methylated, H3K4 was also acetylated in budding yeast and it depends on Gcn5 and Rtt109 (Guillemette et al., 2011). Gcn5 mainly responsible for the acetylation of histone sites H3K9 and H3K14 in yeast and many other pathogenic fungi. Our results showed that *FgGCN5* has a significant impact on acetylation of H3, H3K9, H3K14, H3K18, and H3K27. It was reported that SAS3 plays an important role in H3 acetylation in *M. robertsii* (Fan et al., 2017), and had significant effect on H3K14 acetylation (Lafon et al., 2012). We found that *FgSAS3* affects H3K4ac and H3K14ac. Therefore, we postulate for the first time that acetylation of H3K4 in *F. graminearum* is mainly controlled by *FgSAS3*, and H3K14 can be completely acetylated only by the combined action of *FgSAS3* and *FgGCN5*.

Because conidia and ascospores both act as sources of inoculum for FHB infections, genes associated with reproduction are presumed to be involved in the pathogenicity of *F. graminearum*. The roles of HATs for sexual and asexual development have been extensively studied in many fungal species. For example, in *T. reesei*, the gcn5 mutant strain does not produce conidia (Xin et al., 2013). In *A. nidulans*, the gcnE deletion mutant results in the formation of immature and aberrant conidiophores (Canovas et al., 2014). While in *F. fujikuroi*, no conidia were formed in a GCN5 deletion mutant (Rosler et al., 2016). In this study, we demonstrated that the deletion of *FgGCN5* also results in asexual reproduction failure in *F. graminearum*. Multiple genes control sporulation in *Fusarium* and other fungi, including REN1 (Ohara et al., 2004; Jonkers et al., 2012), AbaA (Son et al., 2013), and FlbC (Jonkers et al., 2012). In *A. nidulans*, the lack of conidiation was caused by the loss of GcnE-mediated H3K9/K14 acetylation at the brlA promoter and reduced the expression of brlA, one of the major activators of conidiation (Adams et al., 1988; Canovas et al., 2014). In this study, we found that the acetylation levels of H3K9 and H3K14 were decreased in Δ*FgGCN5*. Moreover, the transcription factor steA (FGRRES_07310), which has 42% similarity with brlA in *A. nidulans*, was down-regulated in the Δ*FgGCN5* mutant. So *FgsteA* may play a role in GCN5-dependent conidiation. For Δ*FgSAS3*, the size of conidia is larger than that of wild type PH-1. Defects in the cell cycle occurring during conidiogenesis often lead to elongated conidia (Min et al., 2014; Son et al., 2014). The transcription factor *FgwetA* (FGRRES_17727) is involved in conidiogenesis and helps generate longer conidia (Son et al., 2014). But *FgwetA* was not included our DEGs profile. The inappropriate expression of genes involved in cell proliferation and differentiation gave rise to defects in perithecium development in *F. graminearum* (Lin et al., 2011, 2012). The deletion of *FgRTT109, FgSAS3*, or *FgGCN5* resulted in smaller perithecia or even led to the total failure of sexual reproduction. Transcriptome analysis of two deletion mutants showed that *FgSAS3* and *FgGCN5* affected many genes related to sexual development.

In many fungi, the production of secondary metabolites is associated with acetylation modifications. In *A. parasiticus*, the decrease of the acetyltransferase MYST3 led to the reduction of H4 acetylation and blocked aflatoxin production (Roze et al., 2007, 2011). In *A. nidulans*, GcnE can increase the acetylation of H3K9 and H3K14 in the SM cluster, thus inducing the synthesis of orsellinic acid (Nutzmann et al., 2011). Moreover, the absence of TrGcn5 in *T. reesei* significantly affected the acetylation of H3K9 and H3K14 in the cellulase gene promoter cbh1, and the expression of cellulase genes was severely reduced (Xin et al., 2013). The ΔAflgcnE mutant did not produce aflatoxins in *A. flavus* (Lan et al., 2016). In *M. robertsii*, the disruption of a histone acetyltransferase gene Hat1 led to the characterization of 11 new natural products (Fan et al., 2017). In the *F. graminearum* genome, 67 putative SM-associated key genes were predicted (Sieber et al., 2014). Our transcriptome data confirmed that most of these gene clusters are regulated in *FgSAS3* and *FgGCN5*-dependent manners in *F. graminearum*. Furthermore, all genes in the cluster responsible for the biosynthesis of aurofusarin (Malz et al., 2005), are down-regulated in the Δ*FgSAS3* and up-regulated in the Δ*FgGCN5* mutant. These data explain why Δ*FgSAS3* was less colored, while the Δ*FgGCN5* mutant produces more reddish pigment on solid medium compared with WT. The mycotoxin DON has been identified as a critical virulence factor for the infestation of *F. graminearum* (Seong et al., 2008; Boenisch and Schafer, 2011). Both mutants produced little DON compared with PH-1. We also verified that *FgSAS3* and *FgGCN5* affected the expression of genes related to DON biosynthesis. Transcriptome and real-time PCR analysis revealed that all genes in the gene cluster associated with trichothecene biosynthesis are downregulated in both mutants (Figure 6B). These results imply that *FgSAS3* and *FgGCN5* regulate DON biosynthesis in *F. graminearum*.

It is well known that acetylation-induced modifications have a significant impact on the pathogenicity of plant pathogenic fungi. The gcn5 is involved in the pathogenicity to maize plants in *Ustilago maydis* (Gonzalez-Prieto et al., 2014). While, AflGcnE is crucial for maize seed infection (Lan et al., 2016). We observed that in *F. graminearum*, the Δ*FgSAS3* and Δ*FgGCN5* mutants were significantly reduced in pathogenicity. The reduced growth rate of the Δ*FgSAS3* and Δ*FgGCN5* mutants may contribute to its defects in pathogenicity. Virulence-associated DEGs from this two mutants include transcription factors, protein kinases, ABC transporters, regulators of G protein mediated signal transduction, NPRs, and trichothecene mycotoxin biosynthesis genes, which suggest *FgSAS3* and *FgGCN5* are involved in numerous metabolic processes affecting pathogenicity of *F. graminearum*.

The ability of fungi to adapt to environmental stress is critical to their survival. The high-osmolarity glycerol (HOG) pathway plays a vital role in osmoadaptation in *F. graminearum*. HOG pathway-related genes (*FgOs-1, FgOs-2, FgOs-4, FgOs-5*, and *FgRRG-1*) can effectively regulate osmotic stress. Besides, these genes are more sensitive to oxidative stress except *FgOs-1* (Ochiai et al., 2007; Jiang et al., 2011). In our study, Δ*FgSAS3* and Δ*FgGCN5* mutants exhibited increased sensitivity to osmotic stresses as well as to oxidative stress. However, only *FgOs-5* was shown to be significant downregulated in the Δ*FgGCN5* transcriptome data. While several TFs (FGRRES_01555, FGRRES_07075_M, and FGRRES_02531) that influence response to osmotic stress were downregulated in both mutants (Table S5).

Transcription factors are key mediators of signaling pathways and cellular function. TFs phenome database of *F. graminearum* help us to understand the regulatory mechanisms of each TF and provide valuable insight into the changes in gene expression in response to various environmental cues (Son et al., 2011). In our study, many of the transcriptional factors associated with mycelial growth, production of conidia, sexual development, virulence, stress response, and DON biosynthesis were down-regulated in both mutant strains. So we speculate that *FgSAS3* and *FgGCN5* may regulate the expression of the target genes through transcription factors.

In conclusion, we identified four HATs in *F. graminearum* and showed that especially *FgSAS3* and *FgGCN5* are involved in diverse biological processes, including hyphal development, sexual and asexual reproduction, DON biosynthesis, virulence, and stress response. A genome-wide expression analysis revealed a global regulatory impact of *FgSAS3* and *FgGCN5* on gene transcription, probably due to the manifold acetylation targets in H3. These results indicate that the epigenetic modifications influencing chromatin remodeling associated with DON regulation play a crucial role in filamentous fungi. *FgSAS3* and *FgGCN5* may also represent candidate targets for controlling the contamination of crops by *F. graminearum*.

## Author Contributions

HZ, WC, and JF conceived and designed the experiments, reviewed and approved the final manuscript. XK and HZ performed the experiments, analyzed the data, prepared figures and tables and wrote the manuscript. JX and JSX provided assistance during the experiments. AD, TL, and CW wrote and revised drafts of the paper.

### Conflict of interest statement

The authors declare that the research was conducted in the absence of any commercial or financial relationships that could be construed as a potential conflict of interest.

## Supplementary material

Figure S1 Phylogenetic tree analysis of the putative homologs of HATs from several fungal species. Phylogenetic trees of *FgGCN5, FgRTT109, FgSAS2* and *FgSAS3* homologs from several other species were drawn by the divergence distance method using the ClustalW and MEGA 5.0 programs. Neighbor-joining tree with 1000 bootstrap replicates of phylogenetic relationships. *GCN5: F. oxysporum* Fo47 (EWZ52160.1), *A. nidulans* FGSC A4 (AN3621.2), *N. crassa* OR74A (XP_001728480.2), *S. pombe* 972h- (Q9UUK2.1), *S. cerevisiae* S288c (NP_011768.1), *C. albicans* SC5314 (NP_011768.1) *RTT109: F. oxysporum* Fo47 (FOZG_12324), *A. nidulans* FGSC A4 (AN8807.2), *N. crassa* OR74A (NCU09825), *S. pombe* 972h- (Q9Y7Y5.1), *S. cerevisiae* S288c (NP_013099), *C. albicans* SC5314 (XP_718648.1). *SAS2: F. oxysporum* Fo47 (EWZ48415.1), *A. nidulans* FGSC A4 (AN3071.2), *N. crassa* OR74A (XP_959893.1), *S. pombe* 972h- (NP_593736.1), *S. cerevisiae* S288c (NP_013846), *C. albicans* SC5314 (XP_719533.1). *SAS3: F. oxysporum* Fo47 (EWZ39814.1), *A. nidulans* FGSC A4 (AN5640.2), *N. crassa* OR74A (XP_960231.3), *S. pombe* 972h- (NP_593736.1), *S. cerevisiae* S288c (NP_009501.1), *C.albicans* SC5314 (XP_718208.2).

Figure S2 Venn diagram of DEGs in comparisons of Δ*FgSAS3* and Δ*FgGCN5*.

Table S1 Comparisons of the putative HATs in *F. graminearum* with those in other six species.

Table S2 PCR primers used in this study.

Table S3 Differentially expressed genes (DEGs) from Δ*FgSAS3* and Δ*FgGCN5* mutants.

Table S4 Go enrichment of all DEGs.

Table S5 Transcription factors selected from DEGs.

Table S6 DEGs in primary and secondary metabolism profiles.

Table S7 DEGs related to sexual development in two mutants.

Table S8 All virulence-related genes downloaded from the PHI-base database.

Table S9 DEGs related to virulence.
