# Supplementary figures and images for: The Fusarium graminearum Histone Acetyltransferases Are Important for Morphogenesis, DON Biosynthesis, and Pathogenicity
Source: Front Microbiol. 2018 Apr 26;9:654. doi: 10.3389/fmicb.2018.00654 (PMC5932188; doi:10.3389/fmicb.2018.00654)

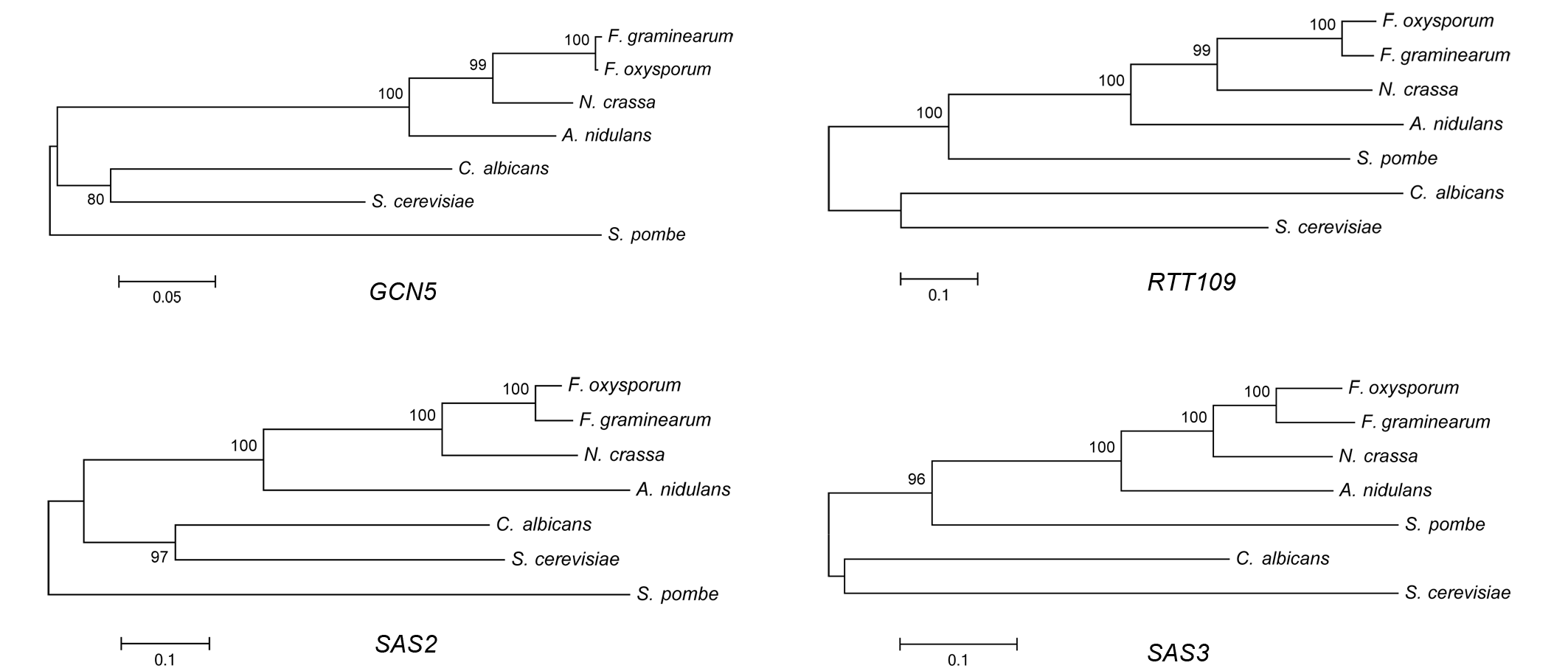

Supplement: Figure S1 — Phylogenetic tree analysis of the putative homologs of HATs from several fungal species. Phylogenetic trees of FgGCN5, FgRTT109, FgSAS2 and FgSAS3 homologs from several other species were drawn by the divergence distance method using the ClustalW and MEGA 5.0 programs. Neighbor-joining tree with 1000 bootstrap replicates of phylogenetic relationships. GCN5: F. oxysporum Fo47 (EWZ52160.1), A. nidulans FGSC A4 (AN3621.2), N. crassa OR74A (XP_001728480.2), S. pombe 972h- (Q9UUK2.1), S. cerevisiae S288c (NP_011768.1), C. albicans SC5314 (NP_011768.1) RTT109: F. oxysporum Fo47 (FOZG_12324), A. nidulans FGSC A4 (AN8807.2), N. crassa OR74A (NCU09825), S. pombe 972h- (Q9Y7Y5.1), S. cerevisiae S288c (NP_013099), C. albicans SC5314 (XP_718648.1). SAS2: F. oxysporum Fo47 (EWZ48415.1), A. nidulans FGSC A4 (AN3071.2), N. crassa OR74A (XP_959893.1), S. pombe 972h- (NP_593736.1), S. cerevisiae S288c (NP_013846), C. albicans SC5314 (XP_719533.1). SAS3: F. oxysporum Fo47 (EWZ39814.1), A. nidulans FGSC A4 (AN5640.2), N. crassa OR74A (XP_960231.3), S. pombe 972h- (NP_593736.1), S. cerevisiae S288c (NP_009501.1), C.albicans SC5314 (XP_718208.2). [file Image_1.TIF]

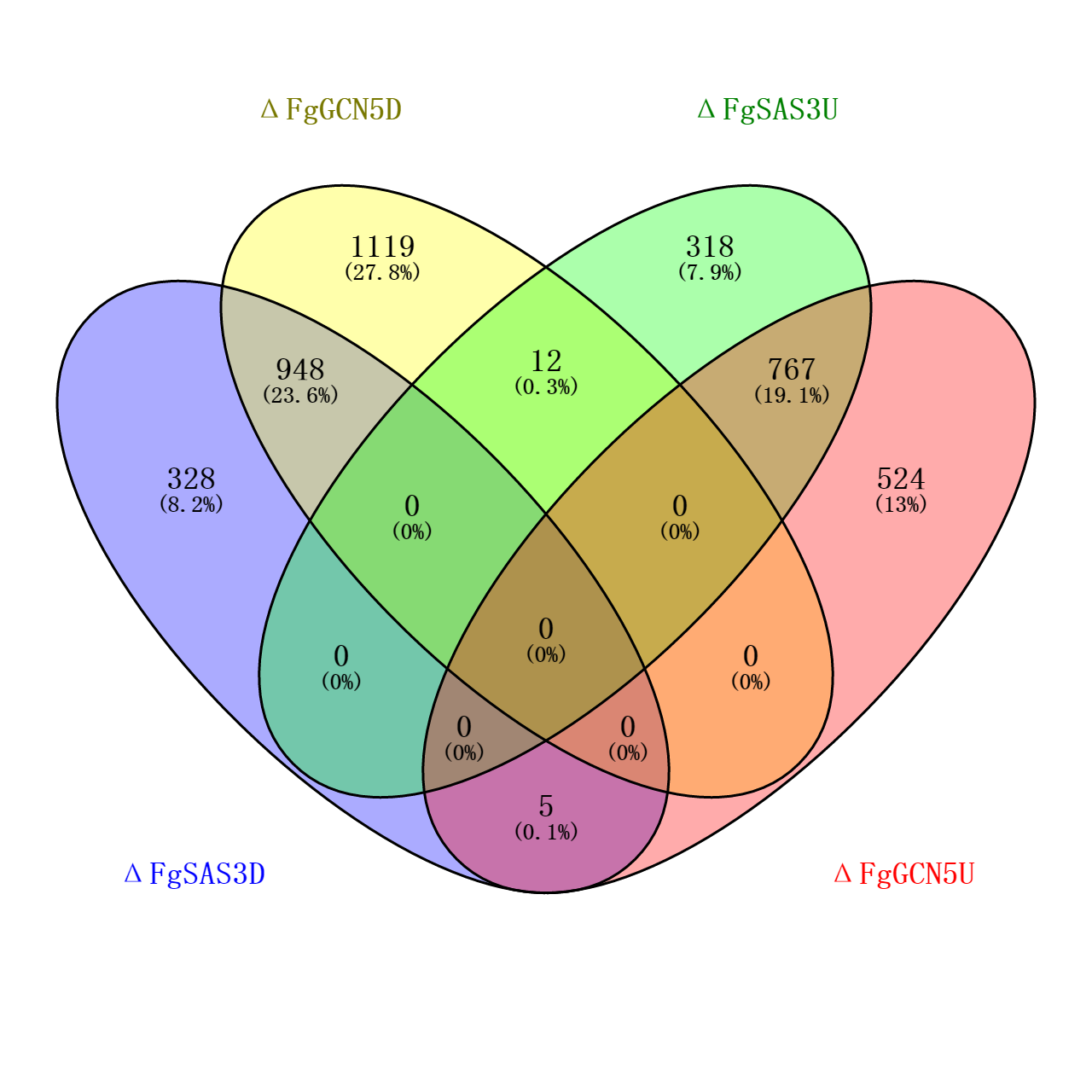

Supplement: Figure S2 — Venn diagram of DEGs in comparisons of ΔFgSAS3 and ΔFgGCN5. [file Image_2.PNG]
