# Supplementary material for: The Fusarium graminearum Histone Acetyltransferases Are Important for Morphogenesis, DON Biosynthesis, and Pathogenicity
Source: Front Microbiol. 2018 Apr 26;9:654. doi: 10.3389/fmicb.2018.00654 (PMC5932188; doi:10.3389/fmicb.2018.00654)
Supplement: Table S1 — Comparisons of the putative HATs in F. graminearum with those in other six species. [file Table_1.docx]

**TABLE S1 |** Comparisons of the putative HATs in *F. graminearum* with those in other six species

|  | GCN5(%) | RTT109(%) | SAS2(%) | SAS3(%) |
| --- | --- | --- | --- | --- |
| *F. oxysporum* | 98 | 84 | 76 | 72 |
| *N. crassa* | 88 | 48 | 58 | 51 |
| *A. nidulans* | 80 | 36 | 37 | 43 |
| *S.cerevisiae* | 66 | 29 | 33 | 38 |
| *S. pombe* | 57 | 35 | 35 | 44 |
| *C. albicans* | 65 | 27 | 31 | 45 |
