# Supplementary material for: The Fusarium graminearum Histone Acetyltransferases Are Important for Morphogenesis, DON Biosynthesis, and Pathogenicity
Source: Front Microbiol. 2018 Apr 26;9:654. doi: 10.3389/fmicb.2018.00654 (PMC5932188; doi:10.3389/fmicb.2018.00654)
Supplement: Table S2 — PCR primers used in this study. [file Table_2.docx]

**TABLE S2 | PCR primers used in this study**

| Oligo name | Sequence |
| --- | --- |
| H850_F | TTCCTCCCTTTATTTCAGATTCAA |
| H850_R | ATGTTGGCGACCTCGTATTGG |
| RTT109_UF | GGTCTTAAUGAGGCATCAACTAATATGGAGAAACA |
| RTT109_UR | GGCATTAAUTGCAGGGGTACCTACTAAGGTACA |
| RTT109_DF | GGACTTAAUAGTTCTGTATAAATACTGTTTGGGTAATGA |
| RTT109_DR | GGGTTTAAUGTGAACGAGGTGAGTAGACTGTCAC |
| RTT109_GF | AAGGGGACGCCCAGTCCTAT |
| RTT109_GR | GTGCTTTGCTCGCTTGATGC |
| RTT109_H850 | CTATTGCATCTCCCGCCGT |
| RTT109_TR | CCCCATCAGTCCCTGTTTTC |
| RTT109_TF | CATTGCTTCTCTTCCTCTTTTC |
| RTT109_H852 | TGTCCTCGTTCCTGTCTGC |
| SAS2_ UF | GGTCTTAAUTTTTCGGCCTATTCGGTTCTGC |
| SAS2_UR | GGCATTAAUCGCTGTGCGACCCTCTTCTCT |
| SAS2_DF | GGACTTAAUCGGCGGTGCCAGGAGC |
| SAS2_DR | GGGTTTAAUTTGAAGCCCCTCGACAATGAC |
| SAS2_GF | TGCTGGTCGTCCGCTTTCA |
| SAS2_GR | TCCTCGTCCCCTCGTGTTTG |
| SAS2_TF | TGAAGAAACACAACGACACGG |
| SAS2_H852 | ACATCGAAGCTGAAAGCACG |
| SAS2_H850 | GGGCAAAGGAATAGAGTAGATG |
| SAS2_TR | AGGGCGGTAGTGGGAAA |
| SAS3_UF | GGTCTTAAUCAGACCATGTGCCAGGCTTGT |
| SAS3_UR | GGCATTAAUGCGCTTGTCGTAATTACTCCCG |
| SAS3_DF | GGACTTAAUATAATTATACCTAACAGCGGGTAGCC |
| SAS3_DR | GGGTTTAAUAGCCCTTTTACGGAATCCATCA |
| SAS3_GF | ATAGTGAGTGTCGCTTGCTTTAGAA |
| SAS3_GR | CAGAATACCGCCGTAGGGTTT |
| SAS3_TF | GTAGGGAGGCAACAAGTGAAA |
| SAS3_H852 | GAAATAAAGGGAGGAAGGGC |
| SAS3_H850 | CAAGGAATCGGTCAATACACTACA |
| SAS3_TR | AATCCAACACCCAACACACAAG |
| GCN5_UF | GGTCTTAAUAAAACGAATACCTTCCTCTGTAATTTG |
| GCN5_UR | GGCATTAAUGATTGGTGCGGGCTCAAC |
| GCN5_DF | GGACTTAAUAGATCTCATCCAGTCAAAAAACTAAAAGC |
| GCN5_DR | GGGTTTAAUAAGGGCCTGACCGGGGATT |
| GCN5_GF | GAGCCACCAGAAAAGAAACC |
| GCN5_GR | ATCACATCGTAATAATCAGCCA |
| GCN5_TF | AAAACGAATACCTTCCTCTGTAATTTG |
| GCN5_H852 | ATGTTGGCGACCTCGTATTGG |
| GCN5_H850 | TTCCTCCCTTTATTTCAGATTCAA |
| GCN5_TR | AAGGGCCTGACCGGGGATT |
| RTT109F+ | GCGTTAATCTAGAACTAGTGAAGAGGGGAGATTGCTGGGTAT |
| RTT109R+ | AGGGAACAAAAGCTGGGTACCTTTTGGGTGAACGAGGTGAGT |
| SAS2F+ | GCGTTAATCTAGAACTAGTGGGCTTCCCGTATCTTGTCTCAG |
| SAS2R+ | AGGGAACAAAAGCTGGGTACGTTACCCTATTCCAATCAACTCCAT |
| SAS3F+ | GCGTTAATCTAGAACTAGTGCCAGTCAGGACAAAGAAGGCA |
| SAS3R+ | AGGGAACAAAAGCTGGGTACCGAGGAGAGTCGGTCAAGGTT |
| Tub_F | AACATGCGTGAGATTGTAAGT |
| Tub_R | TAGTGACCCTTGGCCCAGTTG |
| TRI5_RT_F | TGGGCAAAGGTGTCCAAAGAG |
| TRI5_RT_R | CAATGAGCAGAAAGGTCCAAAATG |
| TRI6_RT_F | CAGCTTATCGCCCTTCCCAC |
| TRI6_RT_R | ATGCCGCCTAAAGTCCCGT |
| TRI10_RT_F | GAAGCGACAGGAGCAAGAACA |
| TRI10_RT_R | CATGATAGAGGCGGCGTAAA |
| TRI12_RT_F | ATCAATCGAGATAACAAGAACGCAG |
| TRI12_RT_R | AAAATGGAGCCAAAAAAGGGG |
